# Supplementary material for: Incomplete Polymerization of Dual-Cured Resin Cement Due to Attenuated Light through Zirconia Induces Inflammatory Responses
Source: Int J Mol Sci. 2023 Jun 7;24(12):9861. doi: 10.3390/ijms24129861 (PMC10298069; doi:10.3390/ijms24129861)
Supplement: Supplementary file 1 [file ijms-24-09861-s001.zip › ijms-2333553-supplementary.pdf]

Supplementary tables

Table S1. High-performance liquid chromatography (HPLC) conditions

Parameter	Condition
Column	Inert Sustain AQ-C18 (GL Science, Tokyo, Japan)
Column dimension	250 mm in length, 4.5 mm in diameter, 3 μm particle size
Mobile phase	75% CH_3OH / 25% H_2O
Flow speed	1.0 mL/min
Column temperature	40°C
Detection wavelength	UV 206 nm
Injection volume	5.0 μL
Run time	10 min

Table S2. Primers used for RT-PCR analyses

Gene	Primers (Fw, forward; Rv, reverse)	Product size (bp)	Accession number
IL-1β	Fw: 5′-TGGAGCAACAAGTGGTGT-3′<br>Rv: 5′-TTGGGATCTACACTCTCCAGC-3′	157	NM_000576.3
IL-6	Fw: 5′-TCAATGAGGAGACTTGCCTG-3′<br>Rv: 5′-GATGAGTTGTCATGTCCTGC-3′	157	NM_001371096.1
PTGES2	Fw: 5′-TCCAGTACCAAAATCGTATTGCT-3′<br>Rv: 5′-AGTGCTTCCAACTCTGCAGACAT-3′	370	NM_000963.4
TNFα	Fw: 5′-GAGGCCAAGCCCTGGTATG-3′<br>Rv: 5′-CGGGCCGATTGATCTCAGC-3′	91	NM_000594.4
MMP2	Fw: 5′-TGAGCTATGGACCTTGGGAGAA-3′<br>Rv: 5′-CCATCGGCGTTCCCATAC-3′	60	NM_001302510.2
MMP9	Fw: 5′-GGACGATGCCTGCAACGT-3′<br>Rv: 5′-CAAATACAGCTGGTTCCCAATCT-3′	64	NM_004994.3
NOS2	Fw: 5′-CAGCGGGATGACTTTCCAA-3′<br>Rv: 5′-AGGCAAGATTTGGACCTGCA-3′	75	NM_000625.4
IL-10	Fw: 5′-TCTCCGAGATGCCTTCAGCAGA-3′<br>Rv: 5′-TCAGACAAGGCTTGGCAACCCA-3′	126	NM_000572.3
ARG1	Fw: 5′-TCGAGGACAGCGAGGCC-3′<br>Rv: 5′-TCGAGGGTGTAGCGTGTAGAGA-3′	85	NM_00111096.1
GAPDH	Fw: 5′-AATCCCATCACCATCTTCCA-3′<br>Rv: 5′-TGGACTCCACGACGTACTCA-3′	82	NM_001357943.2
